# Supramolecular Sandwiches: Halogen-Bonded Coformers Direct [2+2] Photoreactivity in Two-Component Cocrystals

**DOI:** 10.3390/molecules25040907

**Published:** 2020-02-18

**Authors:** Jay Quentin, Dale C. Swenson, Leonard R. MacGillivray

**Affiliations:** Department of Chemistry, University of Iowa, Iowa City, IA 52242, USA; jay-bell@uiowa.edu (J.Q.); dale-swenson@uiowa.edu (D.C.S.)

**Keywords:** cocrystal, crystal engineering, halogen bonding, photodimerization, cyclobutane, pedal motion

## Abstract

The halogen-bond (X-bond) donors 1,3- and 1,4-diiodotetrafluorobenzene (**1,3-di-I-tFb** and **1,4-di-I-tFb**, respectively) form cocrystals with *trans*-1,2-bis(2-pyridyl)ethylene (**2,2′-bpe**) assembled by N···I X-bonds. In each cocrystal, 2(**1,3-di-I-tFb**)·2(**2,2′-bpe**) and (**1,4-di-I-tFb**)·(**2,2′-bpe**), the donor molecules support the C=C bonds of **2,2′-bpe** to undergo an intermolecular [2+2] photodimerization. UV irradiation of each cocrystal resulted in stereospecific and quantitative conversion of **2,2′-bpe** to *rctt*-tetrakis(2-pyridyl)cyclobutane (**2,2′-tpcb**). In each case, the reactivity occurs via face-to-face *π*-stacked columns wherein nearest-neighbor pairs of **2,2′-bpe** molecules lie sandwiched between X-bond donor molecules. Nearest-neighbor C=C bonds are stacked criss-crossed in both cocrystals. The reactivity was ascribed to the olefins undergoing pedal-like motion in the solid state. The stereochemistry of **2,2′-tpcb** is confirmed in cocrystals 2(**1,3-di-I-tFb**)·(**2,2′-tpcb**) and (**1,4-di-I-tFb**)·(**2,2′-tpcb**).

## 1. Introduction

Halogen bonding (X-bonding) is a well-established [1] supramolecular synthon, as this directional, noncovalent force can reliably direct the self-assembly of molecules into supramolecular architectures [2,3]. Electrophilic regions associated with halogen (X) atoms that serve as X-bond donors recognize complementary, nucleophilic (:Nu) functionalities (e.g., N-atoms) on acceptor molecules such that an X-bond, a special class of Lewis acid-base adduct, X···Nu, results [4].

Due to the highly directional character of X-bonds, X-bond donor molecules with rigid geometries have attracted much interest in the field of crystal engineering. A necessary, albeit admittedly ambitious, aim is to achieve a functional level of predictive power in the design of desired geometries and/or topologies based principally on geometries of the X-bond donor and acceptor molecules [2,3]. Successes would be invaluable in the design of functional materials and would constitute a major accomplishment for the fields of crystal engineering and supramolecular chemistry.

In this context, an application of X-bonding of interest to our group is that of exploiting X-bonds as supramolecular synthons to design photoreactive solids. We have devoted efforts to investigate whether multicomponent systems can be assembled into predictable geometries based primarily on the directional character of X-bonds and molecular structures of the components. Ideally, judicious choice of coformers would enable access to solids of functional significance, such as reactivity [5,6,7,8,9].

Perfluorinated iodoarenes are well-established X-bond donors and are currently among the most frequently employed building blocks in the crystal engineering of X-bonded materials. In the context of systematic, geometry-based design, such donors are attractive owing to their planar and rigid structure as well as the well-established enhancement of X-bond donor capability by poly-fluorosubstitution [10]. We very recently described [11] that the cocrystallization of 1,2-diiodotetrafluorobenzene (**1,2-di-I-tFb**) with *trans*-1,2-bis(*n*-pyridyl)ethylene (***n*,*n*’-bpe**, where: *n* = *n*’ = 2, 3, 4) affords cocrystals sustained by N···I X-bonds that make up one-dimensional (1D) and two-dimensional (2D) assemblies. For **2,2′-bpe**, the components generated a discrete aggregate packed as a photostable 1D assembly.

**Scheme 1:**
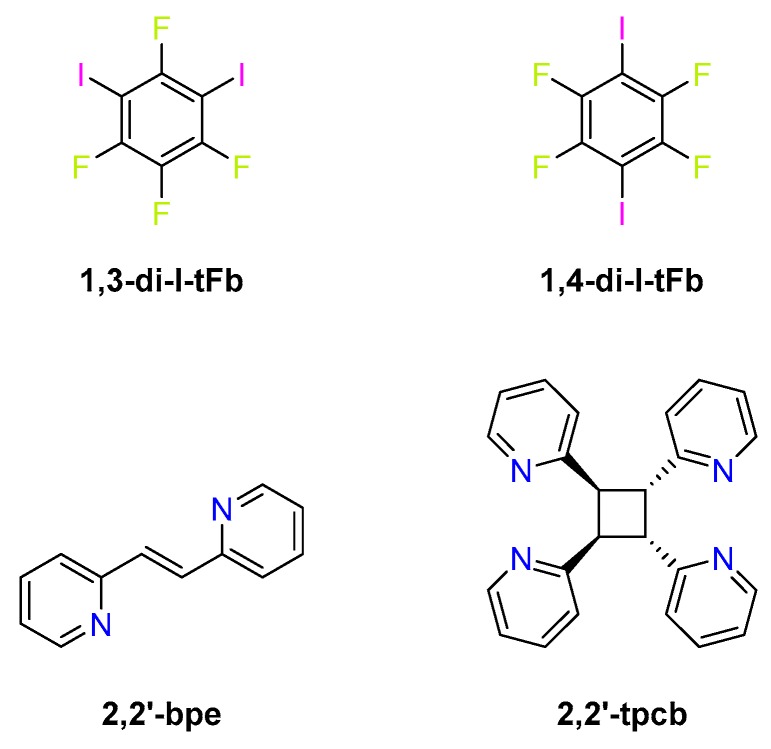
Components for cocrystals.

Among the structural isomers of diiodotetrafluorobenzene, the most ubiquitous X-bond donor is **1,4-di-I-tFb**, with a far less common donor being **1,3-di-I-tFb** (Scheme 1). A survey of the Cambridge Structural Database (CSD) [12] revealed 146 structures that involve **1,4-di-I-tFb** as an X-bond donor, whereas only five structures exist with **1,3-di-I-tFb** as the donor.

**Scheme 2:**
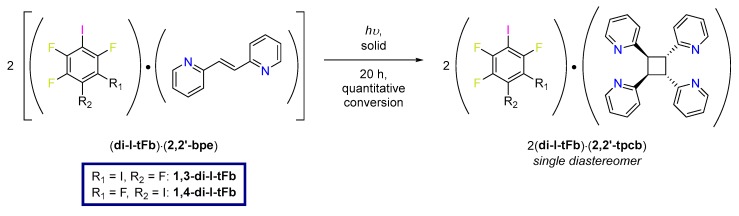
UV-irradiation of 2(**1,3-di-I-tFb**)·2(**2,2′-bpe**) and (**1,4-di-I-tFb**)·(**2,2′-bpe**) to form **2,2′-tpcb**.

Herein, we report cocrystals involving **2,2′-bpe** with **1,3-di-I-tFb** and **1,4-di-I-tFb** as X-bond donors (Scheme 1). The components of each cocrystal, 2(**1,3-di-I-tFb**)·2(**2,2′-bpe**) and (**1,4-di-I-tFb**)·(**2,2′-bpe**), assembled into 1D chains mediated by N···I X-bonds. Adjacent chains interacted via face-to-face *π*-stacking interactions such that two molecules of **2,2′-bpe** lay sandwiched between X-bond donors. We show the arrangements that enabled each sandwiched, nearest-neighbor pair of **2,2′-bpe** to undergo an intermolecular [2+2] photodimerization to furnish *rctt*-tetrakis(2-pyridyl)cyclobutane (**2,2′-tpcb**) quantitatively and stereospecifically (Scheme 2). The solid-state reactivity occurred despite the major fraction of nearest-neighbor C=C bonds of **2,2′-bpe** laying stacked criss-crossed in the solids. We ascribed the reactivity to the C=C bonds undergoing solid-state pedal-like motion [13,14,15,16,17,18,19,20,21,22]. The photoproduct was confirmed in the X-bonded cocrystals 2(**1,3-di-I-tFb**)·(**2,2′-tpcb**) and (**1,4-di-I-tFb**)·(**2,2′-tpcb**).

## 2. Results and Discussion

### 2.1. X-ray Structure of 2(**1,3-di-I-tFb**)·2(**2,2′-bpe**)

**Figure 1:**
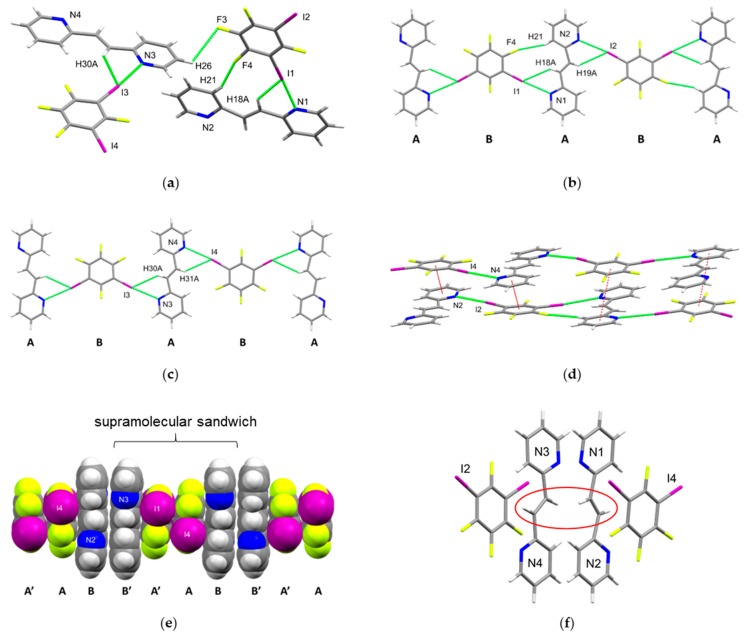
Perspectives of 2(**1,3-di-I-tFb**)·2(**2,2′-bpe**): (**a**) asymmetric unit (olefin disorder omitted for clarity); (**b**) 1D chain based on N1/N2 illustrating ABAB repeat unit; (**c**) 1D chain based on N3/N4 illustrating ABAB repeat unit; (**d**) offset, face-to-face π-stacks (highlighted in red) between chains; (**e**) face-to-face *π*-stacked sandwiches (space-filling) illustrating ABB’A’ repeat unit; and (**f**) *π*-stacked sandwiches highlighting criss-crossed C=C bonds (olefin disorder omitted for clarity).

The components of 2(**1,3-di-I-tFb**)·2(**2,2′-bpe**) crystallized in the orthorhombic, noncentrosymmetric space group *Pna*2_1_ (Table 1). The asymmetric unit consists of two molecules of **1,3-di-I-tFb** and two molecules of **2,2′-bpe** that assemble by a combination of N···I X-bonds (*d*(I1···N1) = 2.96(2) Å; *d*(I2···N2) = 3.00(1) Å; *d*(I3···N3) = 2.99(1) Å; *d*(I4···N4) = 3.04(1) Å) (Table 2), as well as edge-to-edge C-H···I and C-H···F contacts (Figure 1a, Table 3). The pyridyl rings of one molecule of **2,2′-bpe** (N1/N2) lie perfectly coplanar (twist angle = 0.00°), whereas the pyridyl rings of the other (N3/N4) deviate from coplanarity (twist angle ~ 5.35°). Both molecules of **2,2′-bpe** adopt an *anti*-conformation (Figure 1a). The C=C bonds of each molecule of **2,2′-bpe** lie disordered over two sites (occupancies: 72/28 [N1/N2]; 72/28 [N3/N4]). The major fraction (72%) of nearest-neighbor C=C bonds lie criss-crossed [13].

The components formed two crystallographically-unique 1D chains wherein each molecule of **1,3-di-I-tFb** bridges two molecules of **2,2′-bpe** via the N···I X-bonds (Figure 1b,c). Assembly properties of the X-bonded cocrystals 2(**1,3-di-I-tFb**)·2(**2,2′-bpe**), 2(**1,3-di-I-tFb**)·(**2,2′-tpcb**), (**1,4-di-I-tFb**)·(**2,2′-bpe**), and (**1,4-di-I-tFb**)·(**2,2′-tpcb**) are addressed below (Table 3). The chains interact by way of offset, face-to-face *π*-stacks involving **1,3-di-I-tFb** of one chain and one pyridyl (pyr) ring of **2,2′-bpe** of the other chain (*d*(pyr_[N2]_···**1,3-di-I-tFb**_[I3/I4]_ ~ 4.48 Å; *d*(pyr_[N4]_···**1,3-di-I-tFb**_[I1/I2]_ ~ 4.55 Å). Across the *π*-stacked chains, two molecules of **2,2′-bpe** lie sandwiched between two unique molecules of **1,3-di-I-tFb** in an ABB’A’ motif (Figure 1e, Table 3). As a consequence of this arrangement, nearest-neighbor molecules of **2,2′-bpe** stack parallel with C=C bonds separated on the order of 3.64 Å.

Although the C=C bonds of **2,2′-bpe** adopt a criss-crossed geometry, the alkene is photoactive. UV irradiation of a microcrystalline sample of 2(**1,3-di-I-tFb**)·2(**2,2′-bpe**) for a period of 20 h resulted in stereospecific and quantitative conversion of **2,2′-bpe** to **2,2′-tpcb**. The formation of the photoproduct was evidenced by the complete disappearance of the olefinic resonance (7.70 ppm) and emergence of a single cyclobutane resonance (4.93 ppm) [23,24]. We note that, as a single component, **2,2′-bpe** has been reported to be photostable [24].

### 2.2. X-ray Structure of 2(**1,3-di-I-tFb**)·(**2,2′-tpcb**)

**Figure 2:**
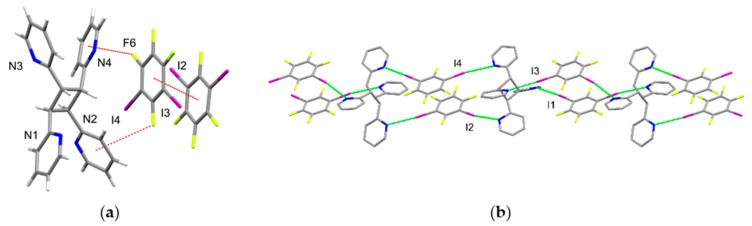
Perspectives of 2(**1,3-di-I-tFb**)·(**2,2′-tpcb**): (**a**) asymmetric unit highlighting *π*···*π* and *π*···F forces (cyclobutane disorder omitted for clarity) and (**b**) 1D chains (H atoms omitted for clarity).

The photoproduct **2,2′-tpcb** interacts with **1,3-di-I-tFb** as a tetratopic [25,26] X-bond acceptor. The components of 2(**1,3-di-I-tFb**)·(**2,2′-tpcb**) crystallize in the monoclinic space group *P*2_1_/*c* (Table 1). The asymmetric unit consists of two unique molecules of **1,3-di-I-tFb** and one molecule of **2,2′-tpcb**. The cyclobutane ring lies disordered over two sites (occupancies: 84/16 at *T* = 150.15 K). The molecules of **1,3-di-I-tFb** interact with each another via face-to-face *π*-stacks (*d* ~ 3.77 Å, Figure 2a). The cyclobutane **2,2′-tpcb** interacts with one molecule of **1,3-di-I-tFb** [I3/I4] via two face-to-face *π*···F forces involving two of the pyridyl (pyr) rings of **2,2′-tpcb** (*d*(pyr_[N1]_···F5) ~ 3.71 Å; *d*(pyr_[N4]_···F6) ~ 3.42 Å, Figure 2a). As a consequence of the assembly process, the components form 1D chains via a combination of N···I X-bonds (*d*(I1···N1) = 2.949(6) Å; *d*(I2···N4) = 3.009(6) Å; *d*(I3···N3) = 2.947(6) Å; *d*(I4···N2) = 3.131(6) Å) (Table 2) and face-to-face π-stacks between molecules of **1,3-di-ItFb** (Figure 2a,b, Table 3).

### 2.3. X-ray Structure of (**1,4-di-I-tFb**)·(**2,2′-bpe**)

**Figure 3:**
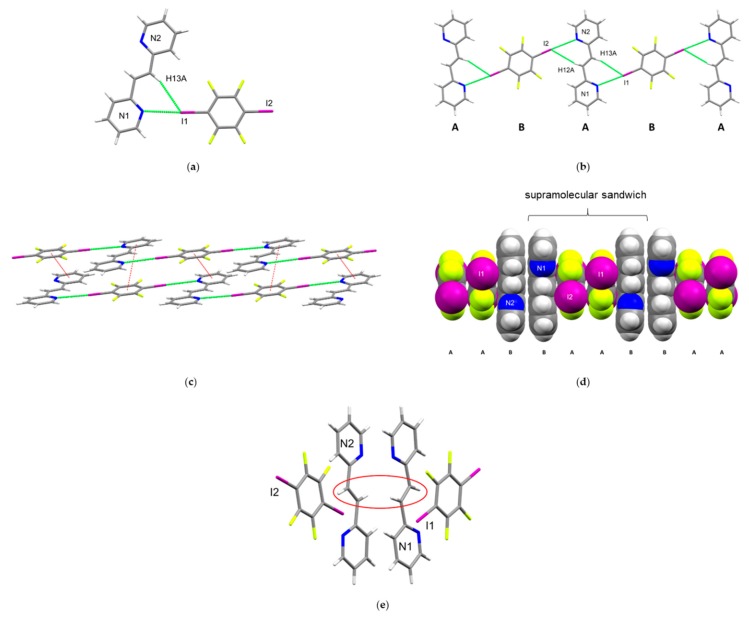
Perspectives of (**1,4-di-I-tFb**)·(**2,2′-bpe**): (**a**) asymmetric unit; (**b**) 1D chain along crystallographic *bc*-plane illustrating the ABAB repeat unit; (**c**) offset, face-to-face *π*-stacks (highlighted in red) between chains; (**d**) face-to-face *π*-stacked sandwiches (space-filling) illustrating the ABBA repeat unit; and (**e**) *π*-stacked sandwiches highlighting criss-crossed C=C bonds (olefin disorder omitted for clarity).

The components of (**1,4-di-I-tFb**)·(**2,2′-bpe**) crystallize in the noncentrosymmetric tetragonal space group *I*4_1_*cd* (Table 1). The asymmetric unit consists of one molecule of **1,4-di-I-tFb** and one molecule of **2,2′-bpe** that assemble by N···I X-bonds (*d*(I1···N1) = 2.967(8) Å; *d*(I2···N2) = 2.982(8) Å) (Figure 3a,b, Table 2). The pyridyl rings of **2,2′-bpe** lie approximately coplanar (twist angle ~ 4.83°). The alkene **2,2′-bpe** adopts an *anti*-conformation (Figure 3a). The C=C bonds of **2,2′-bpe** lie disordered over two sites (occupancies: 66/34). The components form infinite 1D chains along the crystallographic *bc*-plane, sustained by a combination of N···I X-bonds and edge-to-edge C-H···I forces (Table 3). In these chains, each molecule of **1,4-di-I-tFb** bridges two molecules of **2,2′-bpe** by way of two N···I X-bonds such that the chains repeat in an ABAB pattern (Figure 3b, Table 3). Adjacent chains interact by way of offset, face-to-face *π*-stacks involving **1,4-di-I-tFb** and one pyridyl ring of **2,2′-bpe** (*d*(pyr_[N1]_···**1,3-di-I-tFb** ~ 4.67 Å) (Figure 3c).

Similar to 2(**1,3-di-I-tFb**)·2(**2,2′-bpe**), across the *π*-stacked chains, two molecules of **2,2′-bpe** lie sandwiched between two molecules of **1,3-di-I-tFb** in an ABBA motif (Figure 3d, Table 3). As a consequence of this arrangement, nearest-neighbor molecules of **2,2′-bpe** stack parallel with C=C bonds separated on the order of 3.72 Å, with the major sites (occupancies: 66%) of nearest-neighbor C=C bonds being criss-crossed [13,14,15,16,17,18,19,20,21,22]. Despite the crossed geometry, however, **2,2′-bpe** is photoactive. UV irradiation of a microcrystalline sample of (**1,4-di-I-tFb**)·(**2,2′-bpe**) for a period of 20 h resulted in stereospecific and quantitative conversion of **2,2′-bpe** to **2,2′-tpcb**. Formation of the photoproduct was evidenced by complete disappearance of the olefinic resonance (7.70 ppm) and emergence of a single cyclobutane resonance (4.93 ppm) [23,24].

### 2.4. X-ray Structure of (**1,4-di-I-tFb**)·(**2,2′-tpcb**)

**Figure 4:**
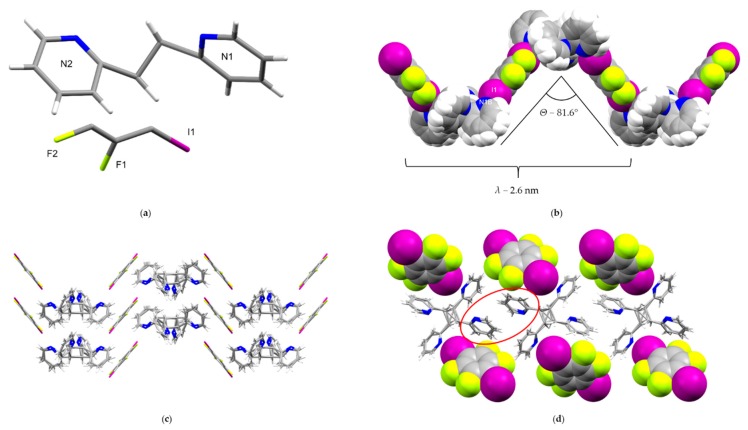
Perspectives of (**1,4-di-I-tFb**)·(**2,2′-tpcb**): (**a**) asymmetric unit (cyclobutane disorder omitted for clarity); (**b**) infinite zig-zag chains (space-filling, view along *c*); (**c**) herringbone pattern resulting from interactions between zig-zag chains; and (**d**) 1D chains of **2,2′-tpcb** generated along the crystallographic *c*-axis, highlighting offset, face-to-face *π*···*π* forces.

The components of (**1,4-di-I-tFb**)·(**2,2′-tpcb**) crystallize in the monoclinic space group *C*2/*c* (Table 1). The asymmetric unit consists of one-half molecule of **1,4-di-I-tFb**, which lies on a crystallographic center of inversion, and one-half molecule of **2,2′-tpcb** which lies on a two-fold axis and is disordered (occupancies: 50/50) by symmetry (Figure 4a). The components of (**1,4-di-I-tFb**)·(**2,2′-tpcb**) assemble primarily via N···I X-bonds (*d*(I1···N1A) = 3.064(4) Å; *d*(I1···N1B) = 2.942(4) Å) to generate infinite zig-zag chains (*λ* ~ 2.6 nm, *Θ* ~ 81.6°) (Figure 4b, Table 3). Adjacent chains interact via C-H···N contacts between pyridyl rings (*d*(C7-H7···N2) = 3.449(8) Å) to generate a herringbone pattern (Figure 4c, Table 3). Adjacent molecules of **2,2′-tpcb** also interact via edge-to-face C-H···*π* forces (*d*(C14-H14···pyr_[N1]_) ~ 4.15 Å) and offset, face-to-face *π*···*π* stacks (*d*(pyr_[N2]_···pyr_[N2]_) ~ 4.51 Å) to generate infinite 1D chains along the crystallographic *c*-axis (Figure 4d).

### 2.5. Photoreactivity

We very recently invoked pedal-like motion to account for the stereospecific and quantitative [2+2] photodimerization of **3,3′-bpe** to form *rctt*-**3,3′-tpcb** in the hydrogen-bonded cocrystal (**catechol**)·(**3,3′-bpe**) [10]. The reactivity occurred between criss-crossed C=C bonds. Pedal motion can also be used to explain the photodimerizations of cocrystals 2(**1,3-di-I-tFb**)·2(**2,2′-bpe**) and (**1,4-di-I-tFb**)·(**2,2′-bpe**) to form *rctt*-**2,2′-tpcb**. The C=C bonds of the two cocrystals also exhibited a criss-crossed geometry that, in contrast to (**catechol**)·(**3,3′-bpe**), also lie disordered. A disordered geometry is typically associated with pedal motion that supports reactivity [11,13,14,15,16,17,18,19,20,21,22].

## 3. Materials and Methods

### 3.1. General Experimental

All reagents and solvents (synthesis grade) were purchased from commercial sources and used as received unless otherwise stated. 1,3-diiodotetrafluorobenzene (**1,3-di-I-tFb**) was purchased from Apollo Scientific; 1,4-diiodotetrafluorobenzene (**1,4-di-I-tFb**) was purchased from Aldrich; *trans*-1,2-bis(2-pyridyl)ethylene (**2,2′-bpe**) was purchased from TCI America; and chloroform (CHCl_3_; certified ACS grade, ≥99.8%, approximately 0.75% EtOH as preservative) was purchased from Fisher Chemical. All cocrystal syntheses were conducted in screw-cap glass scintillation vials. For cocrystal syntheses, “thermal dissolution” refers to the process of combining both solid cocrystal components in a vial, adding solvent portion-wise while maintaining a saturated mixture at room temperature (rt), and tightly capping the vial and heating the mixture on a hot-plate until all solids dissolve to afford a homogeneous solution with the minimum necessary volume of solvent. Compositions of all single crystals were shown to be representative of the bulk material by matching experimental powder X-ray diffraction (pXRD) patterns with those simulated from single-crystal X-ray diffraction (scXRD) data. Yields refer to isolated yields of analytically-pure compounds unless otherwise stated. Melting points (mp) were recorded on samples in open capillary tubes using a manual MEL-TEMP apparatus (Electrothermal Corporation, Staffordshire, UK) and were uncorrected. Photoreactions were conducted in either a NuLink 36 W UV lamp apparatus (λ = 400 nm) or an ACE photocabinet equipped with an ACE quartz, 450 W, broadband, medium pressure, Hg-vapor lamp. Photoreactions were conducted by: (1) grinding single crystals of the cocrystal to a fine powder with an agate mortar and pestle; (2) smearing the powder between two Pyrex plates; and (3) irradiating the powder in 10 h intervals, taking care to ensure uniform irradiation.

### 3.2. Synthetic Procedures

**2(1,3-di-I-tFb)∙2(2,2′-bpe).** Note: 2(**1,3-di-I-tFb**)∙2(**2,2′-bpe**) is photosensitive. Cocrystals of 2(**1,3-di-I-tFb**)∙2(**2,2′-bpe**) were obtained by thermal dissolution of **2,2′-bpe** (85.3 mg, 0.459 mmol) and **1,3-di-I-tFb** (182.4 mg, 0.440 mmol, 0.96 equiv) in CHCl_3_ (2.0 mL). Upon cooling to rt, single crystals of 2(**1,3-di-I-tFb**)∙2(**2,2′-bpe**)—colorless blocks, suitable for scXRD—formed within 6 d. Analytical data: **mp** 90-91 °C (CHCl_3_). **^1^H NMR** (400 MHz, DMSO-*d*_6_): δ 8.61 (ddd, *J* = 4.8, 1.8, 0.9 Hz, 4H_a_), 7.82 (app td, *J* = 7.7, 1.8 Hz, 4H_b_), 7.70 (s, 4H_c_), 7.63 (app dt, *J* = 7.8, 1.0 Hz, 4H_d_), 7.31 (ddd, *J* = 7.5, 4.8, 1.1 Hz, 4H_e_).

**2(1,3-di-I-tFb)∙(2,2′-tpcb).** Single crystals of 2(**1,3-di-I-tFb**)∙2(**2,2′-bpe**) were ground to a fine powder with an agate mortar and pestle and smeared between two Pyrex plates. The plate assembly was placed in an ACE photocabinet. After 20 h, ^1^H NMR assay revealed quantitative and stereospecific conversion to 2(**1,3-di-I-tFb**)·(**2,2′-tpcb**). The product powder was scraped from the plates, dissolved in CHCl_3_ at rt, and allowed to slowly evaporate. Single crystals of 2(**1,3-di-I-tFb**)·(**2,2′-tpcb**)—colorless plates, suitable for scXRD—formed within 9 d. Analytical data: **mp** 112-117 °C, 154–156 °C (dec., CHCl_3_) (two distinct melting points were observed: presumably, one for each of the two coformers. Only the second melting point range (154–156 °C) was accompanied by a noticeable decomposition). **^1^H NMR** (400 MHz, DMSO-*d*_6_): δ 8.34 (ddd, *J* = 4.8, 1.8, 0.8 Hz, 4H_a_), 7.46 (app td, *J* = 7.7, 1.8 Hz, 4H_b_), 7.09 (d, *J* = 7.8 Hz, 4H_c_), 6.99 (ddd, *J* = 7.4, 4.8, 1.1 Hz, 4H_d_), 4.93 (s, 4H_e_).

**(1,4-di-I-tFb)∙(2,2′-bpe).** Note: (**1,4-di-I-tFb**)∙(**2,2′-bpe**) is photosensitive. Cocrystals of (**1,4-di-I-tFb**)∙(**2,2′-bpe**) were obtained by thermal dissolution of **2,2′-bpe** (92.1 mg, 0.495 mmol) and **1,4-di-I-tFb** (208.6 mg, 0.509 mmol, 1.03 equiv) in CHCl_3_ (2.0 mL). Upon cooling to rt, single crystals of (**1,4-di-I-tFb**)∙(**2,2′-bpe**)—colorless plates, suitable for scXRD—formed within 24 h. Analytical data: **mp** 155-156 °C (dec., CHCl_3_). **^1^H NMR** (400 MHz, DMSO-*d*_6_): δ 8.61 (ddd, *J* = 4.8, 1.8, 0.9 Hz, 4H_a_), 7.82 (app td, *J* = 7.7, 1.8 Hz, 4H_b_), 7.70 (s, 4H_c_), 7.63 (app dt, *J* = 7.8, 1.0 Hz, 4H_d_), 7.31 (ddd, *J* = 7.5, 4.8, 1.1 Hz, 4H_e_).

**2(1,4-di-I-tFb)∙(2,2′-tpcb).** Single crystals of 2(**1,4-di-I-tFb**)∙2(**2,2′-bpe**) were ground to a fine powder with an agate mortar and pestle and smeared between two Pyrex plates. The plate assembly was placed in an ACE photocabinet. After 20 h, ^1^H NMR assay revealed quantitative and stereospecific conversion to 2(**1,4-di-I-tFb**)·(**2,2′-tpcb**). Analytical data: **^1^H NMR** (400 MHz, DMSO-*d*_6_): δ 8.34 (ddd, *J* = 4.8, 1.8, 0.8 Hz, 4H_a_), 7.46 (app td, *J* = 7.7, 1.8 Hz, 4H_b_), 7.09 (d, *J* = 7.8 Hz, 4H_c_), 6.99 (ddd, *J* = 7.4, 4.8, 1.1 Hz, 4H_d_), 4.93 (s, 4H_e_).

**(1,4-di-I-tFb)·(2,2′-tpcb).** The product powder, 2(**1,4-di-I-tFb**)·(**2,2′-tpcb**), was scraped from the plate assembly, dissolved in CHCl_3_ at rt, and allowed to slowly evaporate. Single crystals of (**1,4-di-I-tFb**)·(**2,2′-tpcb**)—colorless plates, suitable for scXRD—formed within 6 d. Analytical data: **mp** 174-175 °C (dec., CHCl_3_).

### 3.3. ^1^H NMR Spectroscopy

Proton nuclear magnetic resonance (^1^H NMR) spectra were recorded at room temperature on a Bruker DRX-400 spectrometer at 400 MHz (instrument parameters: field strength: 9.2 T; RF-Console: DRX 3-channel; magnet: shielded superconducting; probe: nature, 5.0 mm BBO-^1^H/^15^N-^31^P; type, double-resonance; temperature range, −100–180 °C). ^1^H NMR data (see Supplementary Materials) are reported as follows: chemical shift (δ, ppm), multiplicity (s = singlet, d = doublet, ddd = doublet of doublet of doublets, app dt = apparent doublet of triplets, app td = apparent triplet of doublets), coupling constant(s) (*J*, Hz), and integration. Chemical shift values were calibrated relative to residual solvent resonance (DMSO: δ_H_ = 2.50 ppm) as the internal standard. All NMR data were collected and plotted within the Bruker TopSpin v4.0.6 software suite.

### 3.4. Powder X-ray Diffraction (pXRD)

Powder X-ray diffraction data were collected at room temperature on a Bruker D8 Advance X-ray diffractometer on samples mounted on glass slides. Each sample was finely ground using an agate mortar and pestle prior to mounting. Instrument parameters: radiation wavelength, CuK*α* (*λ* = 1.5418 Å); scan type, coupled TwoTheta/Theta; scan mode, continuous PSD fast; scan range, 5–40° two-theta; step size, 0.02°; voltage, 40 kV; current, 30 mA. Background subtractions were applied to all experimentally collected data within the Bruker DIFFRAC.EVA v3.1 software suite. All data were plotted in Microsoft Excel 2016. Simulated pXRD patterns were calculated from scXRD data within the Cambridge Crystallographic Data Centre (CCDC) Mercury [27] software suite.

### 3.5. Single-Crystal X-ray Diffraction (scXRD)

Single-crystal X-ray diffraction data were collected on either a Bruker Nonius-Kappa APEX II CCD or a Bruker Nonius-Kappa CCD diffractometer, each equipped with an Oxford Cryosystems 700 series cold N_2_ gas stream cooling system. Data were collected at either room temperature (298.15 K) or low temperature (150.15 K) using graphite-monochromated MoKα radiation (*λ* = 0.71073 Å). Crystals were mounted in Paratone oil on a MiTeGen magnetic mount. Data collection strategies for ensuring maximum data redundancy and completeness were calculated using the Bruker Apex II^TM^ software suite. Data collection, initial indexing, frame integration, Lorentz-polarization corrections, and final cell parameter calculations were likewise accomplished using the Apex II software suite. Multi-scan absorption corrections were performed using SADABS [28]. Structure solution and refinement were accomplished using SHELXT [29] and SHELXL [30], respectively, within the Olex2 [31] graphical user interface. Space groups were unambiguously verified using the PLATON [32] executable. All non-hydrogen atoms were refined anisotropically. All hydrogen atoms were attached via a riding model at calculated positions using suitable HFIX commands. The occupancies of the major and minor positions for the disordered C=C cores within 2(**1,3-di-I-tFb**)·2(**2,2′-bpe**) and (**1,4-di-I-tFb**)·(**2,2′-bpe**) and for the disordered cyclobutane C-C cores within 2(**1,3-di-I-tFb**)·(**2,2′-tpcb**) and (**1,4-di-I-tFb**)·(**2,2′-tpcb**) converged to their respective ratios after each was identified in the difference map and freely refined. Figures of all structures were rendered in the CCDC Mercury [27] software suite.

## 4. Conclusions

We demonstrated that X-bonds support intermolecular [2+2] photodimerizations of **2,2‘-bpe** in the solid state. The reaction occurred stereospecifically and quantitatively to generate **2,2′-tpcb**. The cocrystals 2(**1,3-di-I-tFb**)·2(**2,2′-bpe**) and (**1,4-di-I-tFb**)·(**2,2′-bpe**) provide mounting evidence that X-bonds can be utilized in a more general way to sustain the formation of C-C bonds in solids. Our efforts are now focused on rational approaches to utilize X-bonds to direct stacking and control reactivity in the solid state.

## Figures and Tables

**Table 1:** Crystallographic data and structure refinement statistics *.

Cocrystal	2(1,3-di-I-tFb)·2(2,2′-bpe)	2(1,3-di-I-tFb)·(2,2′-tpcb)	(1,4-di-I-tFb)·(2,2′-bpe)	(1,4-di-I-tFb)·(2,2′-tpcb)
CCDC deposition number	1940759	1967637	1940762	1967634
Empirical formula	C_18_H_10_F_4_I_2_N_2_	C_36_H_20_F_8_I_4_N_4_	C_18_H_10_F_4_I_2_N_2_	C_30_H_20_F_4_I_2_N_4_
Formula weight/g·mol^−1^	584.08	1168.16	584.08	766.3
Temperature/K	296.15	150.15	296.15	296.15
Crystal system	orthorhombic	monoclinic	tetragonal	monoclinic
Space group	*Pna*2_1_	*P*2_1_/c	*I*4_1_*cd*	*C*2/*c*
*a*/Å	21.975(2)	9.3925(9)	14.1404(14)	25.422(3)
*b*/Å	19.2247(19)	18.8849(19)	14.1404(14)	5.4525(5)
*c*/Å	9.0190(9)	20.596(2)	38.138(4)	21.183(2)
*α*/°	90	90	90	90
*β*/°	90	90.337(5)	90	112.099(5)
*γ*/°	90	90	90	90
Volume/Å^3^	3810.2(6)	3653.2(6)	7625.7(17)	2720.5(5)
Z	8	4	16	4
*ρ*_calc_/g·cm^−3^	2.036	2.124	2.035	1.871
*μ*/mm^−1^	3.342	3.486	3.34	2.367
*F*(000)	2192	2192	4384	1480
Crystal size/mm^3^	0.32 × 0.23 × 0.22	0.205 × 0.145 × 0.03	0.23 × 0.19 × 0.045	0.16 × 0.16 × 0.05
Radiation	MoKα (*λ* = 0.71073)	MoKα (*λ* = 0.71073)	MoKα (*λ* = 0.71073)	MoKα (*λ* = 0.71073)
2*Θ* range for data collection/°	3.706 to 53.308	4.844 to 52.798	5.762 to 53.438	6.326 to 53.426
Index ranges	−27 ≤ h ≤ 27, −24 ≤ k ≤ 23, −11 ≤ l ≤ 11	−11 ≤ h ≤ 11, −23 ≤ k ≤ 23, −25 ≤ l ≤ 25	−17 ≤ h ≤ 17, −17 ≤ k ≤ 17, −48 ≤ l ≤ 41	−32 ≤ h ≤ 32, −6 ≤ k ≤ 6, −26 ≤ l ≤ 21
Reflections collected	24085	41568	21996	7854
Independent reflections	7967 [*R*_int_ = 0.0302, *R*_sigma_ = 0.0287]	7413 [*R*_int_ = 0.0373, *R*_sigma_ = 0.0299]	3771 [*R*_int_ = 0.0374, *R*_sigma_ = 0.0225]	2870 [*R*_int_ = 0.0338, *R*_sigma_ = 0.0413]
Data/restraints/parameters	7967/1/482	7413/4/498	3771/1/242	2870/1/196
Goodness-of-fit on *F*^2^	1.091	1.153	1.062	1.077
Final *R* indices [*I* ≥ 2*σ* (*I*)]	*R*_1_ = 0.0537	*R*_1_ = 0.0463	*R*_1_ = 0.0260	*R*_1_ = 0.0352
	*wR*_2_ = 0.1355	*wR*_2_ = 0.0864	*wR*_2_ = 0.0552	*wR*_2_ = 0.0745
*R* indices (all data)	*R*_1_ = 0.0685	*R*_1_ = 0.0613	*R*_1_ = 0.0394	*R*_1_ = 0.0641
	*wR*_2_ = 0.1522	*wR*_2_ = 0.0926	*wR*_2_ = 0.0603	*wR*_2_ = 0.0838
Largest diff. peak/hole/e·Å^−3^	3.50/−1.09	3.41/−3.00	0.24/−0.34	0.441/−0.676
Flack parameter	0.284(14)	-	0.254(13)	-

* $R_{1}\;=\;\sum \left|\left|F_{o}\right|\;-\;\left|F_{c}\right|\right|/\sum \left|F_{o}\right|$, $wR_{2}\;=\;\left[\sum w{\left(F_{o}^{2}\;-\;F_{c}^{2}\right)}^{2}\right]/\sum {\left[w{\left(F_{o}^{2}\right)}^{2}\right]}^{1/2}$. Goodness-of-fit on *F*^2^ $=\;{\left[\sum w{\left(\left|F_{o}\right|-\left|F_{c}\right|\right)}^{2}/\left(N_{\mathrm{obs}}-N_{\mathrm{parameter}}\right)\right]}^{1/2}$.

**Table 2:** X-bond metrics for cocrystals.

Cocrystal	X-Bond	*d*(N···I)/Å	*Θ*(C-I···N)/°	X-Bond Type	Prs *
2(**1,3-di-I-tFb**)·(**2,2′-bpe**)	I1···N1	2.96(2)	172.64	I	16
	I2···N2	3.00(1)	174.16	I	15
	I3···N3	2.99(1)	172.24	I	15
	I4···N4	3.04(1)	174.42	I	14
2(**1,3-di-I-tFb**)·(**2,2′-tpcb**)	I1···N1	2.949(6)	174.39	I	16
	I2···N4	3.009(6)	167.06	I	15
	I3···N3	2.947(6)	177.44	I	17
	I4···N2	3.131(6)	169.48	I	11
(**1,4-di-I-tFb**)·(**2,2′-bpe**)	I1···N1	2.967(8)	177.09	I	16
	I2···N2	2.982(8)	177.16	I	16
(**1,4-di-I-tFb**)·(**2,2′-tpcb**)	I1···N1A	3.064(4)	174.36	I	13
	I1···N1B	2.942(4)	171.42	I	17

* Prs ≡ percent relative shortening = {1 − *d*(N···I)/[*r*_vdW_(I) + *r*_vdW_(N)]}·100, with *r*_vdW_(N) = 1.55 Å, and *r*_vdW_(I) = 1.98 Å.

**Table 3:** Structural features of cocrystals.

Cocrystal	Primary Assembly	Secondary Assembly	Photoreactivity
2(**1,3-di-I-tFb**)·2(**2,2′-bpe**)	two unique, infinite 1D chains based on N···I	face-to-face ABB’A’ π-stacked sandwiches	active
2(**1,3-di-I-tFb**)·(**2,2′-tpcb**)	infinite 1D chain based on N···I and face-to-face *π*-stacks	-	-
(**1,4-di-I-tFb**)·(**2,2′-bpe**)	infinite 1D chain based on N···I and edge-to-edge C-H···I	face-to-face ABBA π-stacked sandwiches	active
(**1,4-di-I-tFb**)·(**2,2′-tpcb**)	infinite zig-zag chains based on N···I	herringbone pattern based on C-H···N	-

## Supplementary Materials

The following are available online, Figure S1: ^1^H NMR spectrum of **2,2′-bpe**, Figure S2: ^1^H NMR spectrum of 2(**1,3-di-I-tFb**)·(**2,2′-tpcb**), Figure S3: ^1^H NMR spectrum of 2(**1,4-di-I-tFb**)·(**2,2′-tpcb**), Figure S4: pXRD pattern of 2(**1,3-di-I-tFb**)·2(**2,2′-bpe**), Figure S5: pXRD pattern of 2(**1,3-di-I-tFb**)·(**2,2′-tpcb**), Figure S6: pXRD pattern of (**1,4-di-I-tFb**)·(**2,2′-bpe**), Figure S7: pXRD pattern of (**1,4-di-I-tFb**)·(**2,2′-tpcb**), Tables S1–S4: Crystallographic data and structure refinement statistics for 2(**1,3-di-I-tFb**)·2(**2,2′-bpe**), **2**(**1,3-di-I-tFb**)·(**2,2′-tpcb**), (**1,4-di-I-tFb**)·(**2,2′-bpe**), and (**1,4-di-I-tFb**)·(**2,2′-tpcb**), Table S5: Bond metrics for cocrystals, Table S6: Melting point data for cocrystals and coformers thereof.
