# Supplementary material for: Supramolecular Sandwiches: Halogen-Bonded Coformers Direct [2+2] Photoreactivity in Two-Component Cocrystals
Source: Molecules. 2020 Feb 18;25(4):907. doi: 10.3390/molecules25040907 (PMC7070622; doi:10.3390/molecules25040907)
Supplement: Supplementary file 1 [file molecules-25-00907-s001.pdf]

Supporting Information

Supramolecular Sandwiches: Halogen-Bonded Coformers Direct [2+2] Photoreactivity in Two-Component Cocrystals

Jay Quentin, Dale C. Swenson, and Leonard R. MacGillivray*

Department of Chemistry, University of Iowa, Iowa City, IA, 52242-1294, USA.
*E-mail: len-macgillivray@uiowa.edu

ORCID

Jay Quentin: 0000-0002-1729-7774

Leonard R. MacGillivray: 0000-0003-0875-677X

Supporting Information:

S1. Synthetic Procedures
2(**1,3-di-I-tFb**)·2(**2,2'-bpe**).
2(**1,3-di-I-tFb**)·(**2,2'-tpcb**).
(**1,4-di-I-tFb**)·(**2,2'-bpe**).
2(**1,4-di-I-tFb**)·(**2,2'-tpcb**).
(**1,4-di-I-tFb**)·(**2,2'-tpcb**).

S2. Nuclear Magnetic Resonance (NMR) Spectroscopy Data
Figure S1. ^{1}H NMR spectrum of **2,2'-bpe**.
Figure S2. ^{1}H NMR spectrum of 2(**1,3-di-I-tFb**)·(**2,2'-tpcb**).
Figure S3. ^{1}H NMR spectrum of 2(**1,4-di-I-tFb**)·(**2,2'-tpcb**).

S3. Powder X-Ray Diffraction (pXRD) Data
Figure S4. pXRD pattern of 2(**1,3-di-I-tFb**)·2(**2,2'-bpe**).
Figure S5. pXRD pattern of 2(**1,3-di-I-tFb**)·(**2,2'-tpcb**).
Figure S6. pXRD pattern of **(1,4-di-I-tFb**)·(**2,2'-bpe**).
Figure S7. pXRD pattern of **(1,4-di-I-tFb**)·(**2,2'-tpcb**).

S4. Single-crystal X-Ray Diffraction (scXRD) Data
Table S1. Crystallographic data and structure refinement statistics for 2(**1,3-di-I-tFb**)·2(**2,2'-bpe**).
Table S2. Crystallographic data and structure refinement statistics for **2(1,3-di-I-tFb**)·(**2,2'-tpcb**).
Table S3. Crystallographic data and structure refinement statistics for **(1,4-di-I-tFb**)·(**2,2'-bpe**).
Table S4. Crystallographic data and structure refinement statistics for **(1,4-di-I-tFb**)·(**2,2'-tpcb**).

S5. Bond Metrics and Melting Point Tables
Table S5. Bond metrics for cocrystals.
Table S6. Melting point data for cocrystals and coformers thereof.

S6. References

S1. Synthetic Procedures

Materials and Methods. All reagents and solvents (synthesis grade) were purchased from commercial sources and used as received unless otherwise stated. 1,3-diiodotetrafluorobenzene (**1,3-di-I-tFb**) was purchased from Apollo Scientific©; 1,4-diiodotetrafluorobenzene (**1,4-di-I-tFb**) was purchased from Aldrich©; *trans*-1,2-bis(2-pyridyl)ethylene (**2,2'-bpe**) was purchased from TCI America©; All cocrystal syntheses were conducted in

screw-cap glass scintillation vials. For cocrystal syntheses, "thermal dissolution' refers to the process of: combining both solid cocrystal components in a vial; adding solvent portion-wise while maintaining a saturated mixture at rt; tightly capping the vial and heating the mixture on a hot-plate until all solids dissolve to afford a homogeneous solution with the minimum necessary volume of solvent. Compositions of all single crystals were shown to be representative of the bulk material by matching experimental pXRD patterns with those simulated from scXRD data. Yields refer to isolated yields of analytically pure compounds unless otherwise stated. Melting points were recorded on samples in open capillary tubes using a manual MEL-TEMP® apparatus (Electrothermal™ Corporation) and are uncorrected. Photoreactions were conducted in either a NuLink 36 W UV lamp apparatus (λ = 400 nm) or an ACE® photocabinet equipped with an ACE® quartz, 450 W, broadband,* medium pressure, Hg-vapor lamp. Photoreactions were conducted by: 1) grinding single crystals of the cocrystal to a fine powder with an agate mortar and pestle; 2) smearing the powder between two Pyrex® plates; 3) irradiating the powder in 10 h intervals, taking care to ensure uniform irradiation.†

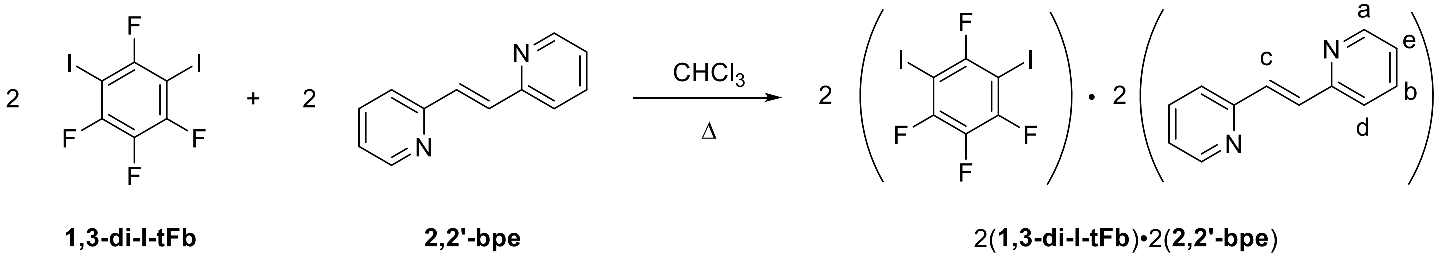

2(1,3-di-I-tFb)·2(2,2'-bpe). <u>Note</u>: 2(**1,3-di-I-tFb**)·2(**2,2'-bpe**) is photosensitive. Cocrystals of 2(**1,3-di-I-tFb**)·2(**2,2'-bpe**) were obtained by thermal dissolution of **2,2'-bpe** (85.3 mg, 0.459 mmol) and **1,3-di-I-tFb** (182.4 mg, 0.440 mmol, 0.96 equiv) in $CHCl_3$ (2.0 mL). Upon cooling to rt, single crystals of 2(**1,3-di-I-tFb**)·2(**2,2'-bpe**)—colorless blocks, suitable for scXRD—formed within 6 d. Analytical data: **mp** 90-91 °C ($CHCl_3$). **^{1}H NMR** (400 MHz, DMSO-d_6): δ 8.61 (ddd, J = 4.8, 1.8, 0.9 Hz, $4H_a$), 7.82 (app td, J = 7.7, 1.8 Hz, $4H_b$), 7.70 (s, $4H_c$), 7.63 (app dt, J = 7.8, 1.0 Hz, $4H_d$), 7.31 (ddd, J = 7.5, 4.8, 1.1 Hz, $4H_e$).

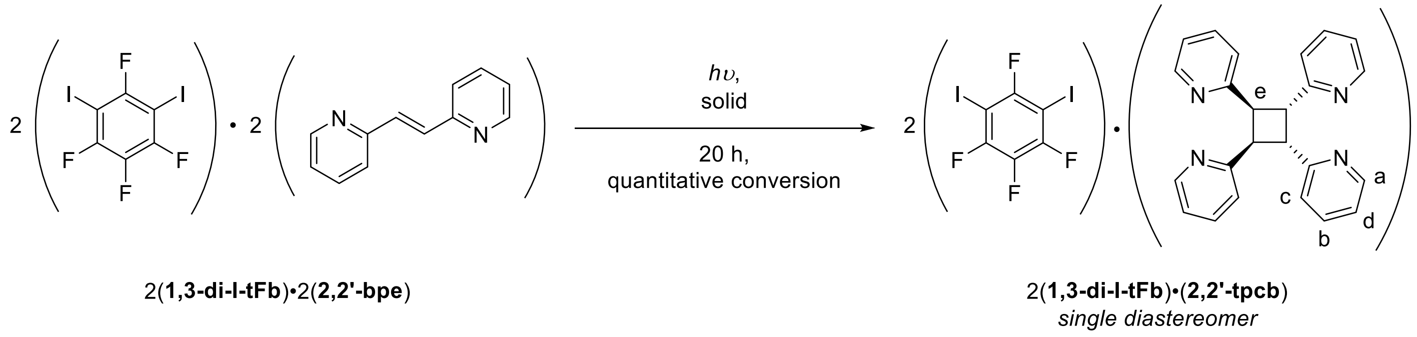

2(1,3-di-I-tFb)·(2,2'-tpcb). Single crystals of 2(**1,3-di-I-tFb**)·2(**2,2'-bpe**) were ground to a fine powder with an agate mortar and pestle and smeared between two Pyrex® plates. The plate assembly was placed in an ACE® photocabinet. After 20 h, ^{1}H NMR assay revealed quantitative and stereospecific conversion to 2(**1,3-di-I-tFb**)·(**2,2'-tpcb**). The product powder was scraped from the plates, dissolved in $CHCl_3$ at rt, and allowed to slowly evaporate: single crystals of 2(**1,3-di-I-tFb**)·(**2,2'-tpcb**)—colorless plates, suitable for scXRD—formed within 9 d. Analytical data: **mp** 112-117 °C, 154-156 °C (dec., $CHCl_3$)‡. **^{1}H NMR** (400 MHz, DMSO-d_6): δ 8.34 (ddd, J = 4.8, 1.8, 0.8 Hz, $4H_a$), 7.46 (app td, J = 7.7, 1.8 Hz, $4H_b$), 7.09 (d, J = 7.8 Hz, $4H_c$), 6.99 (ddd, J = 7.4, 4.8, 1.1 Hz, $4H_d$), 4.93 (s, $4H_e$).

---

* λ = 1367.3-222.4 nm. Of the total energy emitted by the broadband lamp, approximately 40-48% is in the ultraviolet portion of the spectrum, 40-43% in the visible, and the balance in the infrared.

† Uniform irradiation of cocrystal powders was ensured by: occasionally scraping (razor blade) the powder from each plate assembly; combining powders from all plate assemblies; homogenizing the combined, bulk powder *via* thorough grinding (agate mortar and pestle); redistributing the homogenized powder among each plate assembly. Each plate assembly was also flipped between irradiation intervals to ensure equal irradiation of both faces of the plate assembly.

‡ Two distinct melting points were observed: presumably, one for each of the two coformers. Only the second melting point range (154-156 °C) was accompanied by noticeable decomposition.

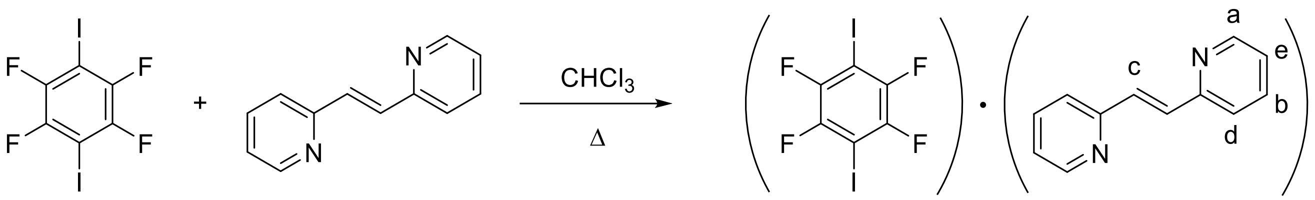

1,4-di-I-tFb **2,2'-bpe** (**1,4-di-I-tFb**)•(**2,2'-bpe**)

(1,4-di-I-tFb)·(2,2’-bpe). Note: (**1,4-di-I-tFb**)·(**2,2’-bpe**) is photosensitive. Cocrystals of (**1,4-di-I-tFb**)·(**2,2’-bpe**) were obtained by thermal dissolution of **2,2’-bpe** (92.1 mg, 0.495 mmol) and **1,4-di-I-tFb** (208.6 mg, 0.509 mmol, 1.03 equiv) in $CHCl_3$ (2.0 mL). Upon cooling to rt, single crystals of (**1,4-di-I-tFb**)·(**2,2’-bpe**)—colorless plates, suitable for scXRD—formed within 24 h. Analytical data: **mp** 155-156 °C (dec., $CHCl_3$). **^{1}H NMR** (400 MHz, DMSO-d_6): δ 8.61 (ddd, J = 4.8, 1.8, 0.9 Hz, 4H_a), 7.82 (app td, J = 7.7, 1.8 Hz, 4H_b), 7.70 (s, 4H_c), 7.63 (app dt, J = 7.8, 1.0 Hz, 4H_d), 7.31 (ddd, J = 7.5, 4.8, 1.1 Hz, 4H_e).

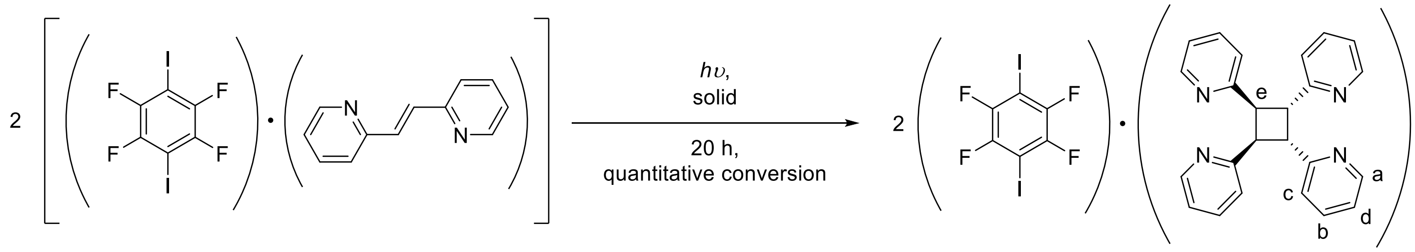

(**1,4-di-I-tFb**)•(**2,2'-bpe**)

2(**1,4-di-I-tFb**)•(**2,2'-tpcb**)
single diastereomer

2(1,4-di-I-tFb)·(2,2’-tpcb). Single crystals of 2(**1,4-di-I-tFb**)·2(**2,2’-bpe**) were ground to a fine powder with an agate mortar and pestle and smeared between two Pyrex® plates. The plate assembly was placed in an ACE® photocabinet. After 20 h, ^{1}H NMR assay revealed quantitative and stereospecific conversion to 2(**1,4-di-I-tFb**)·(**2,2’-tpcb**). Analytical data: **^{1}H NMR** (400 MHz, DMSO-d_6): δ 8.34 (ddd, J = 4.8, 1.8, 0.8 Hz, 4H_a), 7.46 (app td, J = 7.7, 1.8 Hz, 4H_b), 7.09 (d, J = 7.8 Hz, 4H_c), 6.99 (ddd, J = 7.4, 4.8, 1.1 Hz, 4H_d), 4.93 (s, 4H_e).

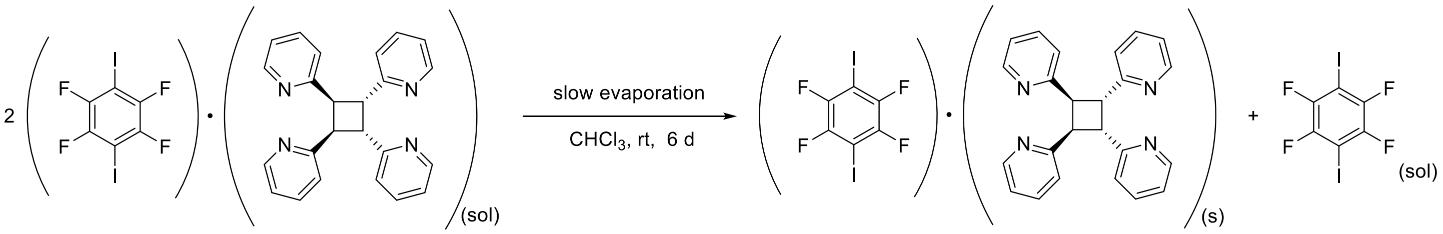

2(**1,4-di-I-tFb**)•(**2,2'-tpcb**) (**1,4-di-I-tFb**)•(**2,2'-tpcb**) **1,4-di-I-tFb**

(1,4-di-I-tFb)·(2,2’-tpcb). The product powder, 2(**1,4-di-I-tFb**)·(**2,2’-tpcb**), was scraped from the plate assembly, dissolved in $CHCl_3$ at rt, and allowed to slowly evaporate: single crystals of (**1,4-di-I-tFb**)·(**2,2’-tpcb**)—colorless plates, suitable for scXRD—formed within 6 d. Analytical data: **mp** 174-175 °C (dec., $CHCl_3$).

S2. NMR Spectra

Proton nuclear magnetic resonance (^{1}H NMR) spectra were recorded at room temperature on a Bruker® DRX-400 spectrometer at 400 MHz (instrument parameters: field strength: 9.2 T; RF-Console: DRX 3-channel; magnet: shielded superconducting; probe: nature, 5.0 mm BBO-^{1}H/^{15}N-^{31}P; type, double-resonance; temperature range, -100-180 °C). ^{1}H NMR data are reported as follows: chemical shift (δ, ppm), multiplicity (s = singlet, d = doublet, ddd = doublet of doublet of doublets, app dt = apparent doublet of triplets, app td = apparent triplet of doublets), coupling constant(s) (J, Hz), and integration. Chemical shift values were calibrated relative to residual solvent resonance (DMSO: δ_H = 2.50 ppm) as the internal standard. All NMR data were collected and plotted within the Bruker® TopSpin™ v4.0.6 software suite.

"JQB 2,2'-bpe (DMSO)"

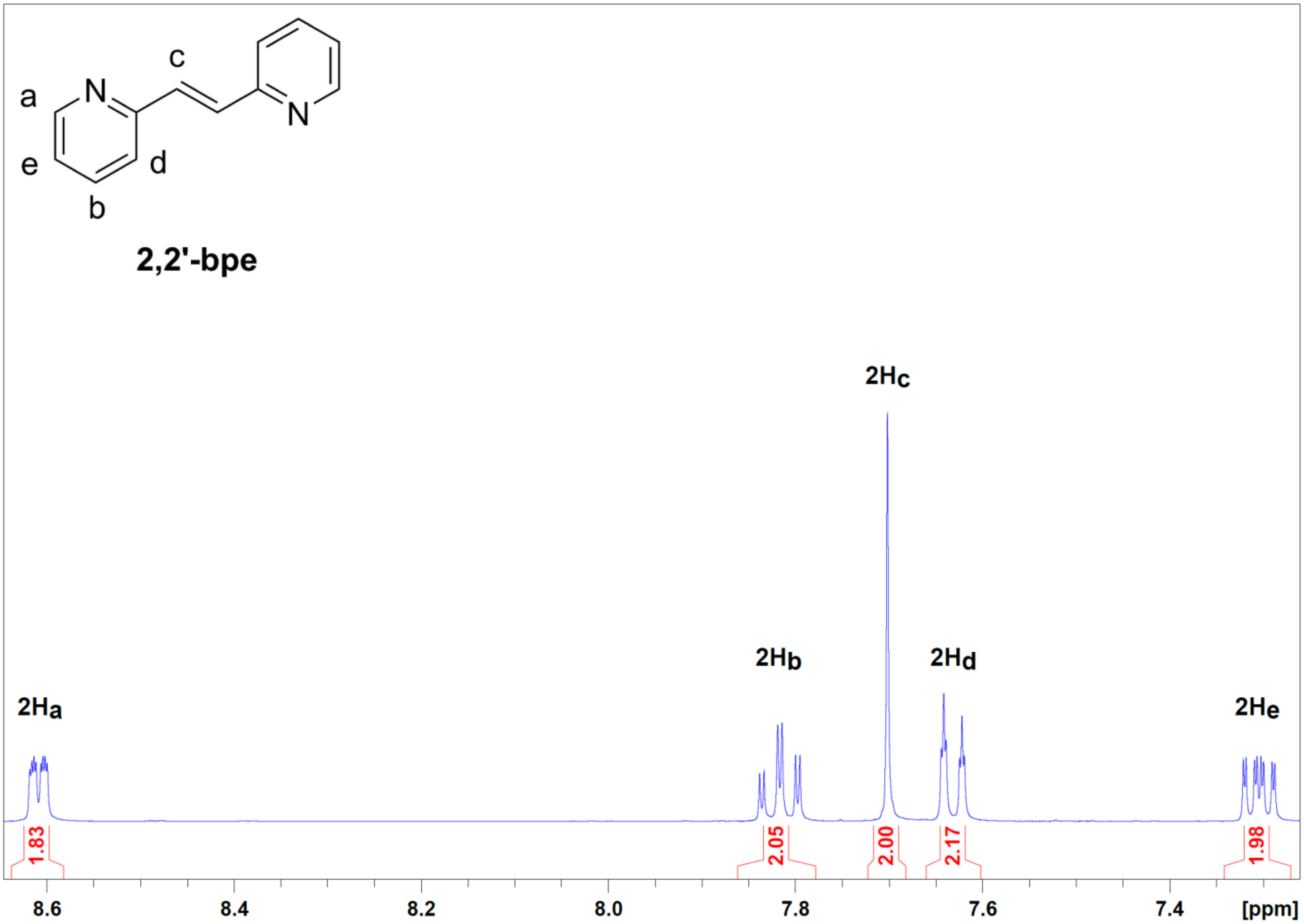

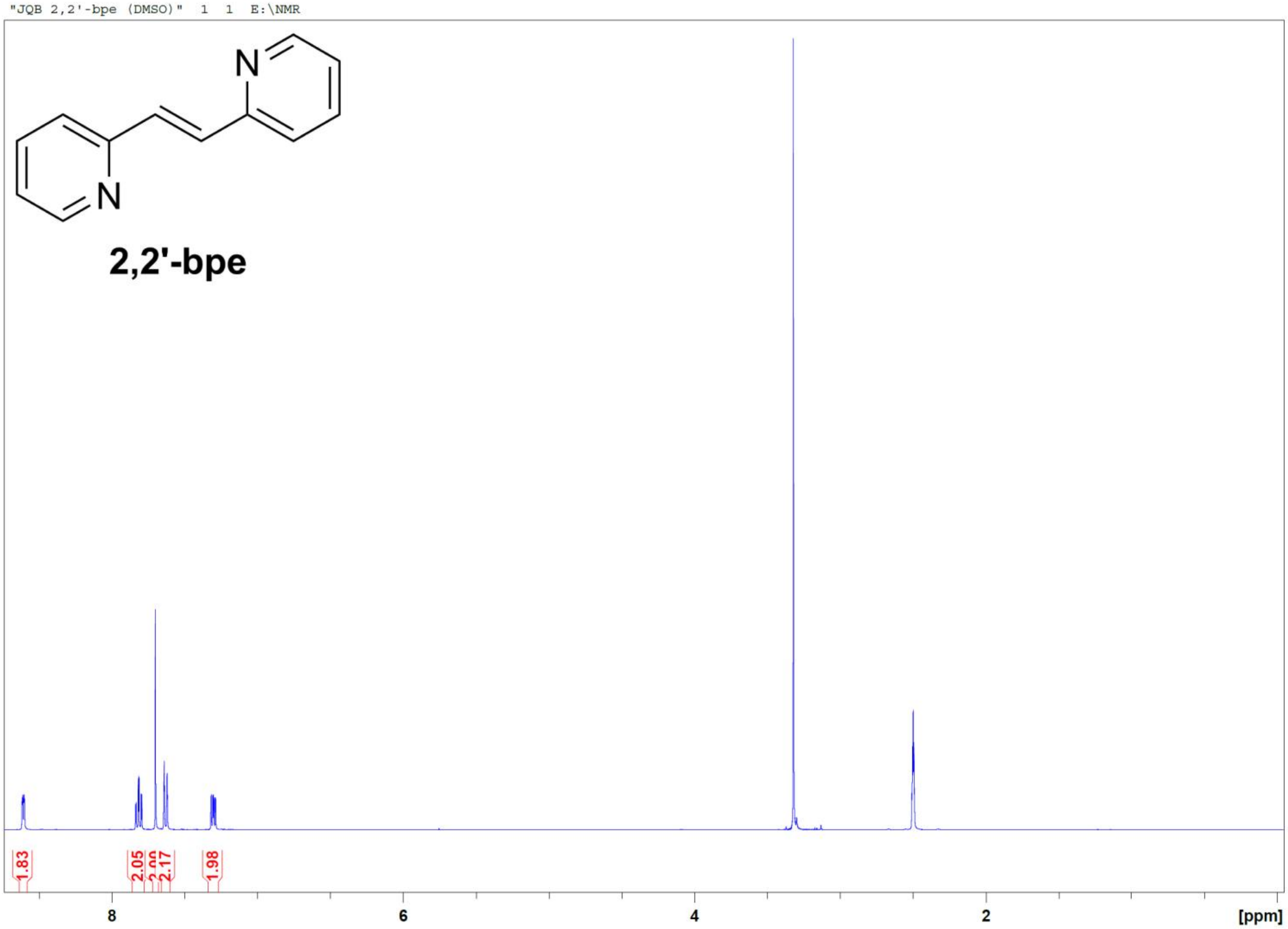

Figure S1. ^{1}H NMR (400 MHz, DMSO-d_6) spectrum of **2,2'-bpe**. The horizontal axis is calibrated relative to DMSO.

"JQB-3-279 20 h"

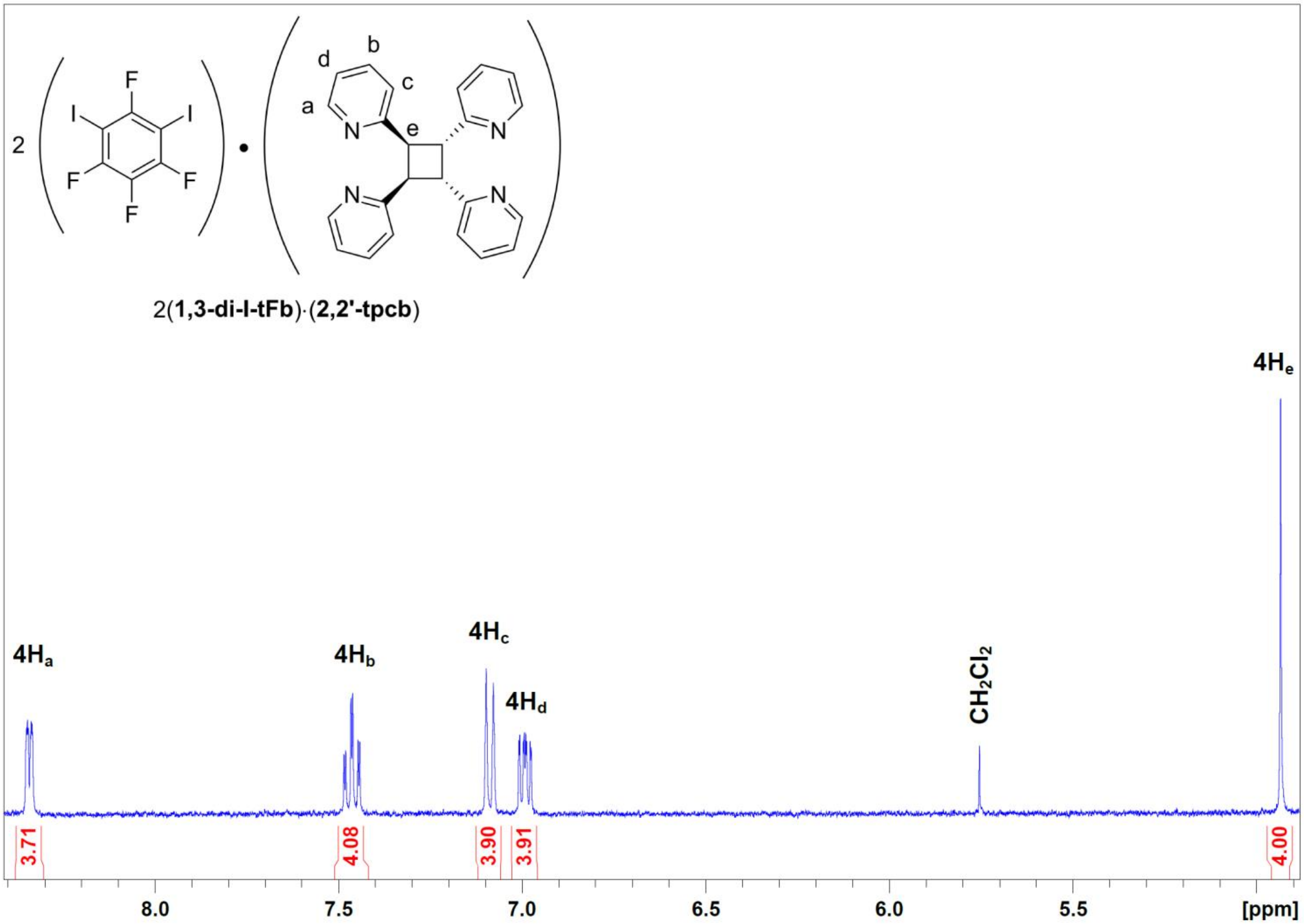
2(1,3-di-I-tFb)·(2,2'-tpcb)
4Ha
4Hb
4Hc
4Hd
CH2Cl2
4He
3.71
4.08
3.90
3.91
4.00
8.0
7.5
7.0
6.5
6.0
5.5
[ppm]

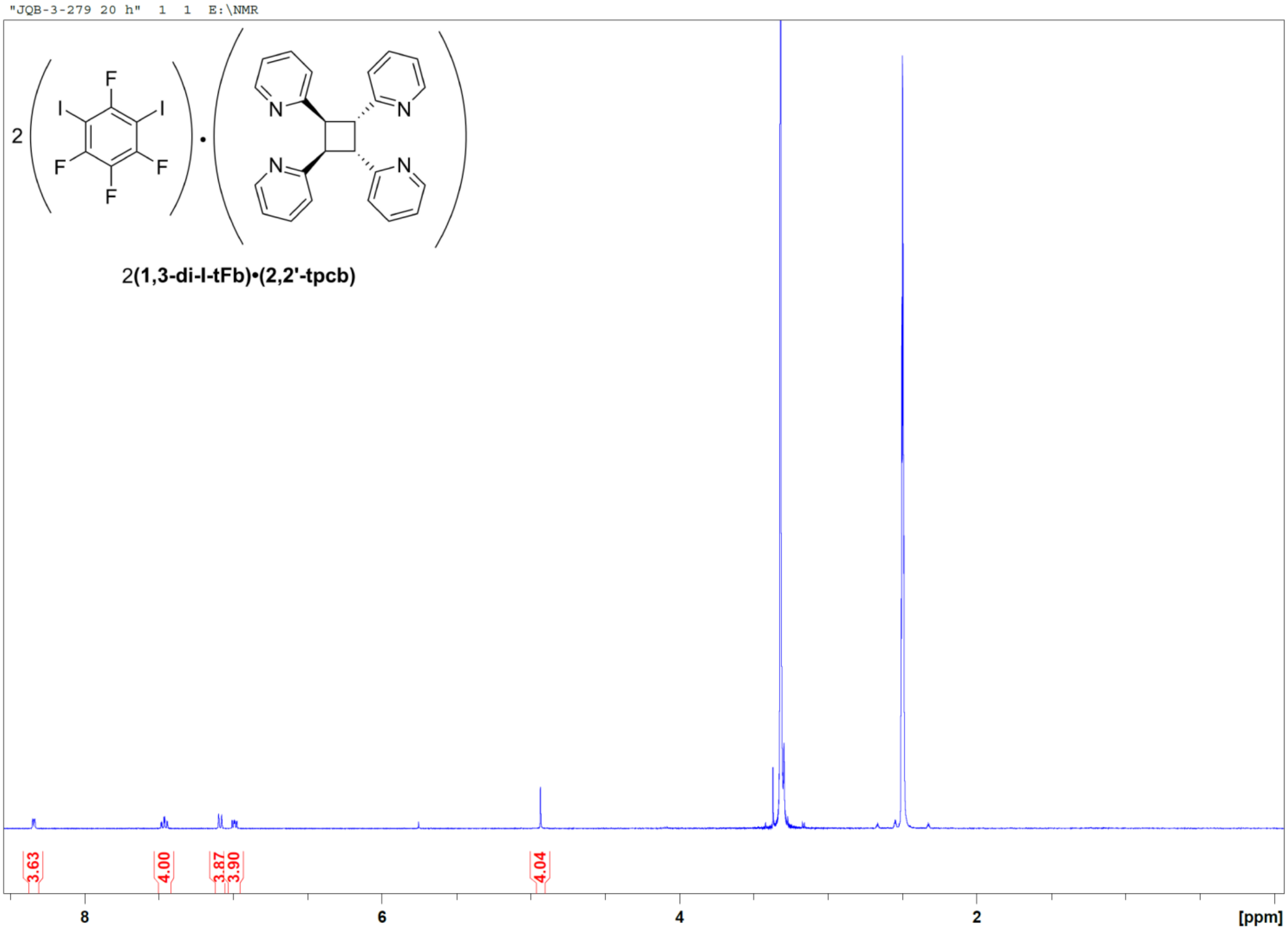

Figure S2. ^{1}H NMR (400 MHz, DMSO-d_6) spectrum of 2(**1,3-di-I-tFb**)·(**2,2'-tpcb**). The horizontal axis is calibrated relative to DMSO. Note the absence of the olefinic resonance at 7.70 ppm (H_c of **2,2'-bpe**).

"JQB-3-257 20 h"

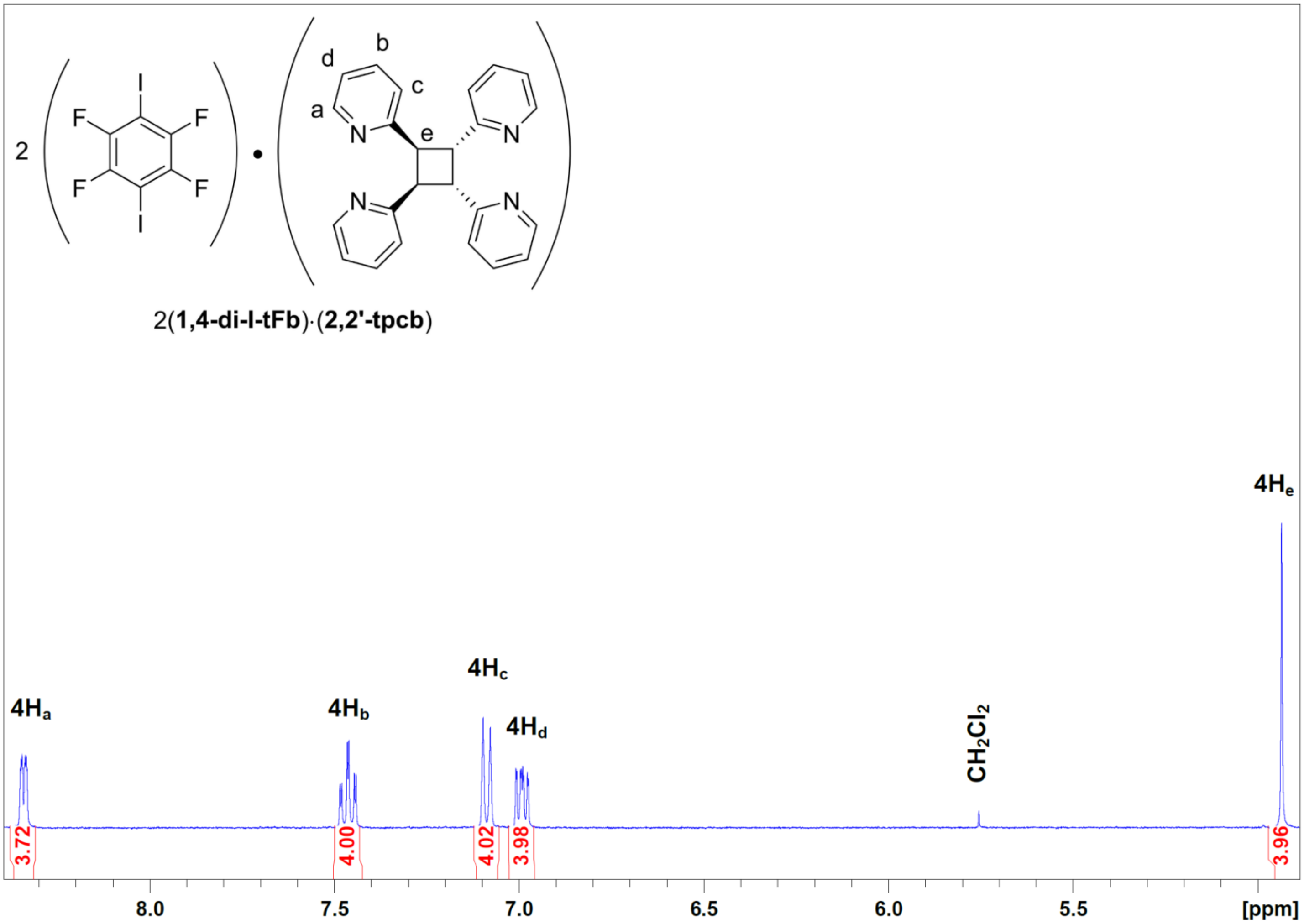
2(1,4-di-I-tFb)·(2,2'-tpcb)
4Ha
4Hb
4Hc
4Hd
CH2Cl2
4He
3.72
4.00
4.02
3.98
3.96
8.0
7.5
7.0
6.5
6.0
5.5
[ppm]

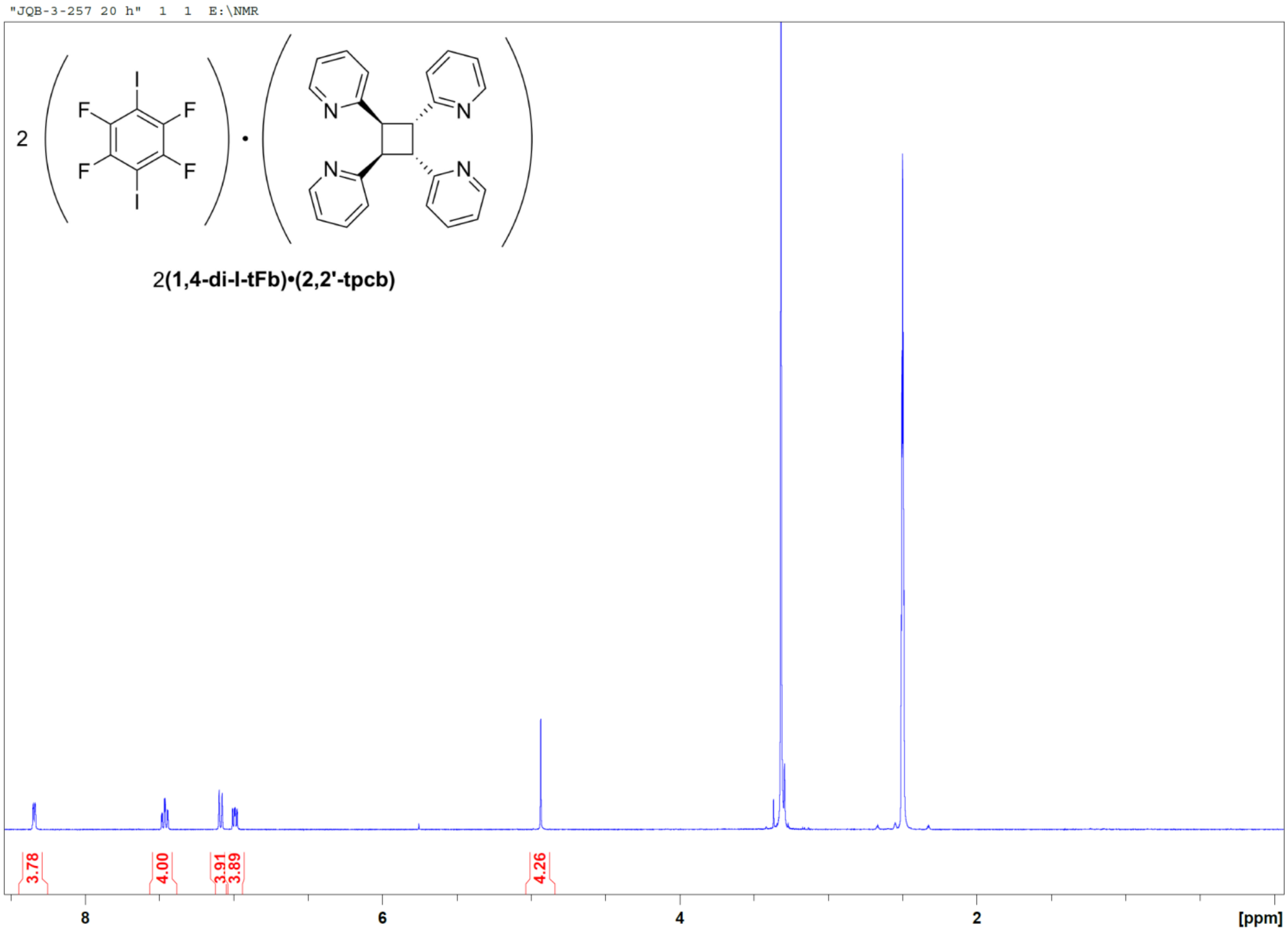

Figure S3. ^{1}H NMR (400 MHz, DMSO-d_6) spectrum of 2(**1,4-di-I-tFb**)·(**2,2'-tpcb**). The horizontal axis is calibrated relative to DMSO. Note the absence of the olefinic resonance at 7.70 ppm (H_c of **2,2'-bpe**).

S3. pXRD Data

Powder X-ray diffraction data were collected at room temperature on a Bruker® D8 Advance X-ray diffractometer on samples mounted on glass slides. Each sample was finely ground using an agate mortar and pestle prior to mounting. Instrument parameters: radiation wavelength, CuKα (λ = 1.5418 Å); scan type, coupled TwoTheta/Theta; scan mode, continuous PSD fast; scan range, 5-40° two-theta; step size, 0.02°; voltage, 40 kV; current, 30 mA. Background subtractions were applied to all experimentally collected data within the Bruker® DIFFRAC.EVA v3.1 software suite. All data were plotted in Microsoft® Excel 2016. Simulated pXRD patterns were calculated from scXRD data within the CCDC Mercury[1] software suite.

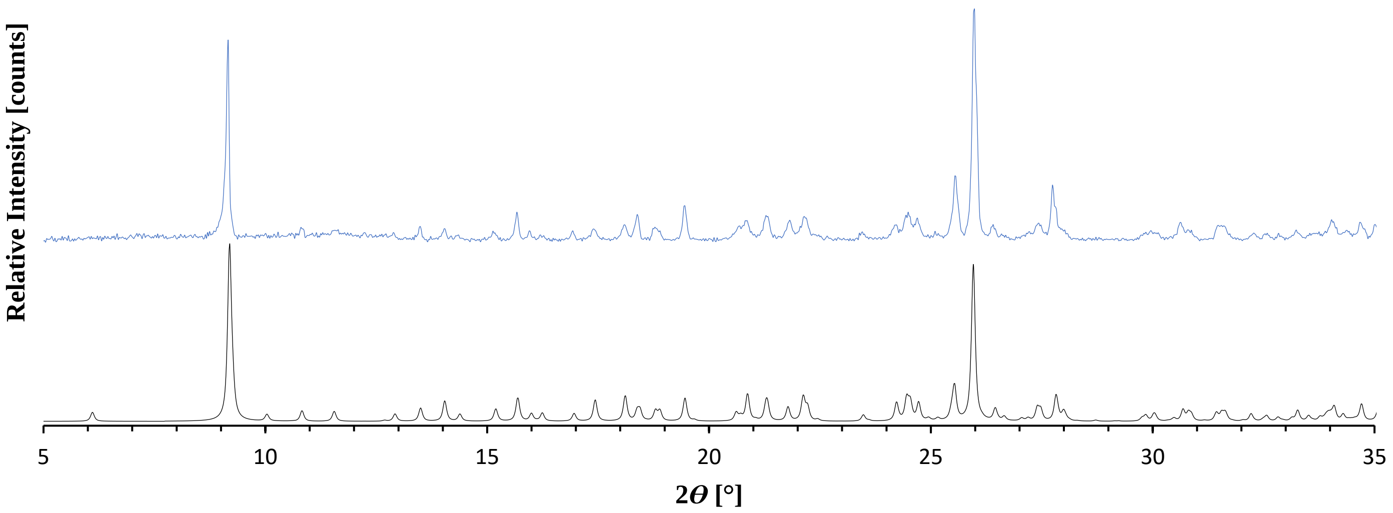

Figure S4. Experimental (top) and simulated (bottom) pXRD patterns for 2(**1,3-di-I-tFb**)·2(**2,2’-bpe**).

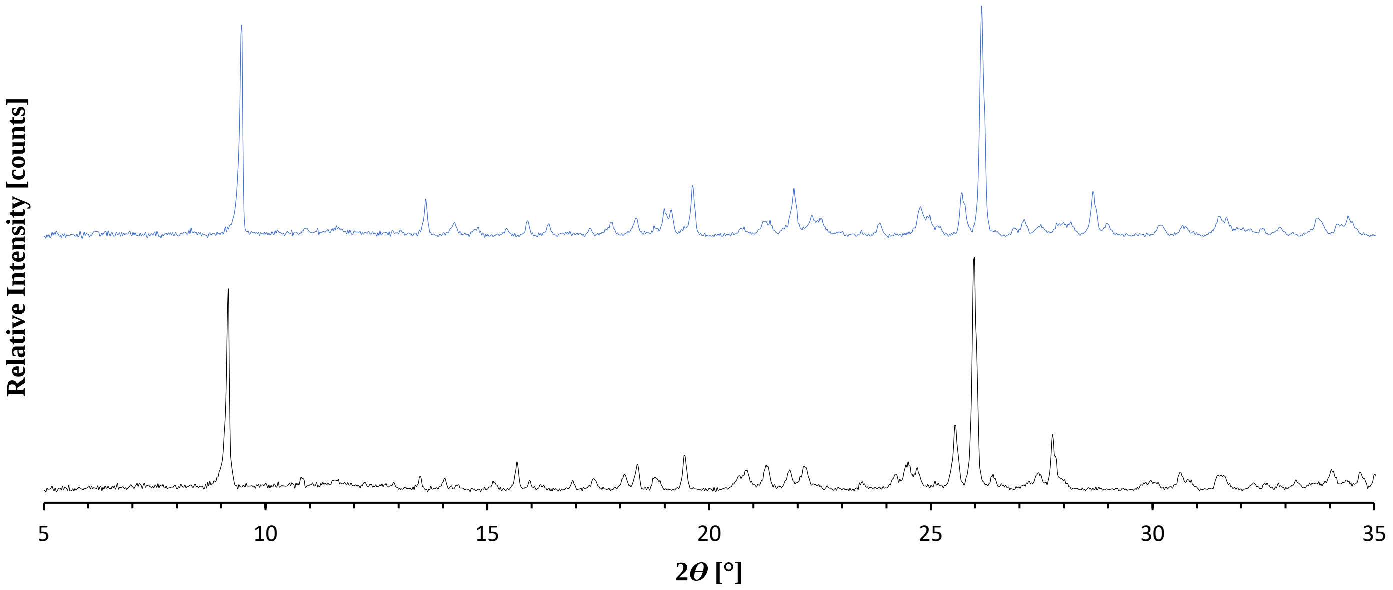

Figure S5. Experimental (top) and simulated (bottom) pXRD patterns for 2(**1,3-di-I-tFb**)·(**2,2’-tpcb**).

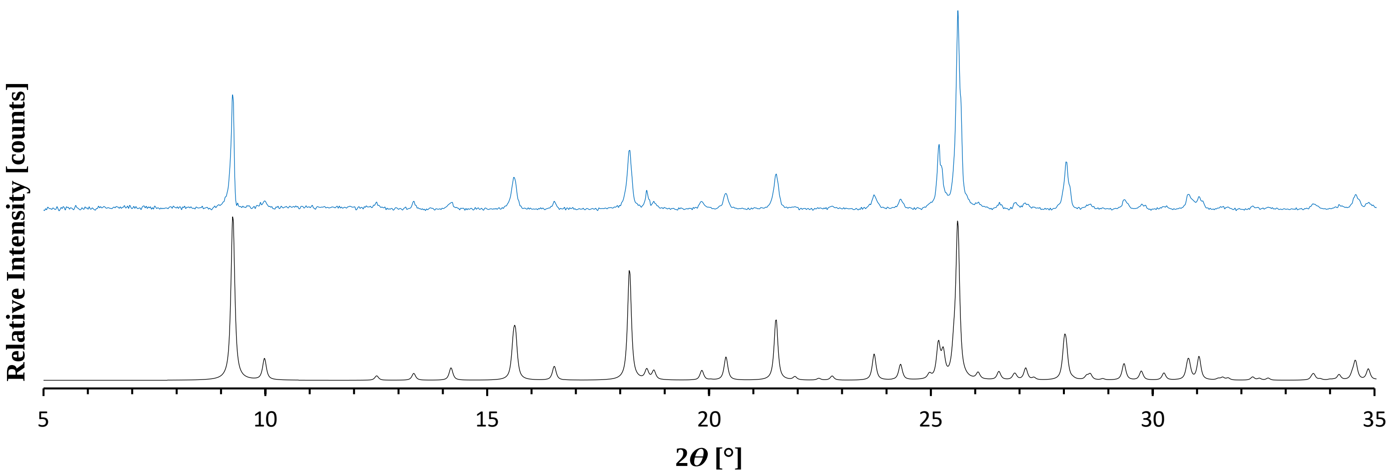

Figure S6. Experimental (top) and simulated (bottom) pXRD patterns for (**1,4-di-I-tFb**)·(**2,2'-bpe**).

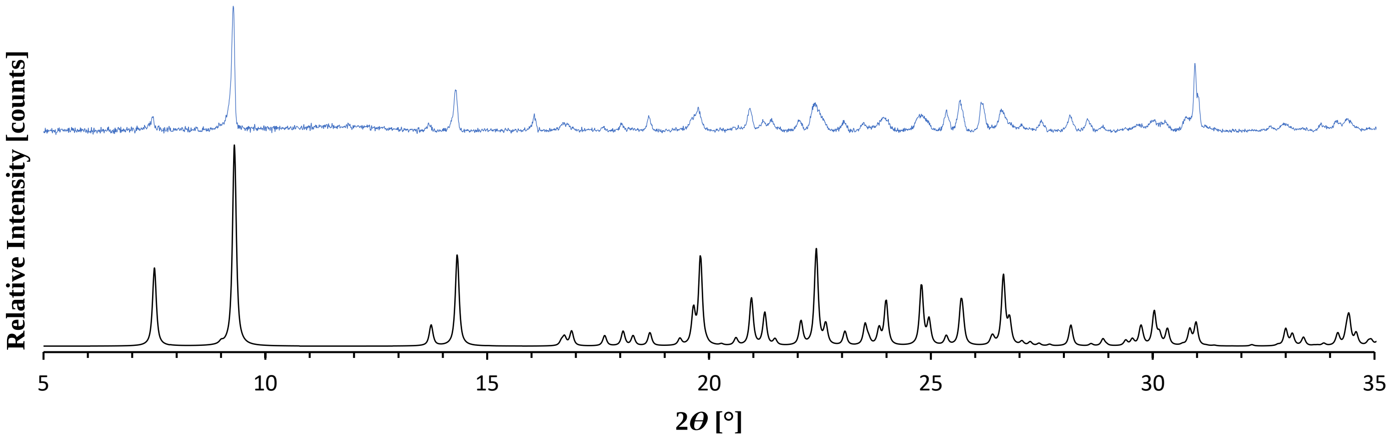

Figure S7. Experimental (top) and simulated (bottom) pXRD patterns for (**1,4-di-I-tFb**)·(**2,2'-tpcb**).

S4. scXRD Data

Single-crystal X-ray diffraction data were collected on either a Bruker® Nonius-Kappa© APEX II CCD or a Bruker® Nonius-Kappa© CCD diffractometer, each equipped with an Oxford Cryosystems® 700 series cold N_2 gas stream cooling system. Data were collected at either room temperature (298.15 K) or low temperature (150.15 K) using graphite-monochromated MoKα radiation (λ = 0.71073 Å). Crystals were mounted in Paratone® oil on a MiTeGen© magnetic mount. Data collection strategies for ensuring maximum data redundancy and completeness were calculated using the Bruker® Apex II™ software suite. Data collection, initial indexing, frame integration, Lorentz-polarization corrections and final cell parameter calculations were likewise accomplished using the Apex II software suite. Multi-scan absorption corrections were performed using SADABS.[2] Structure solution and refinement were accomplished using SHELXT[3] and SHELXL,[4] respectively, within the Olex2[5] graphical user interface. Space groups were unambiguously verified using the PLATON©[6] executable. All non-hydrogen atoms were refined anisotropically. All hydrogen atoms were attached *via* a riding model at calculated positions using suitable HFIX commands. The occupancies of the major and minor positions for the disordered C=C cores within 2(**1,3-di-I-tFb**)·2(**2,2'-bpe**) and (**1,4-di-I-tFb**)·(**2,2'-bpe**) and for the disordered cyclobutane C-C cores within 2(**1,3-di-I-tFb**)·(**2,2'-tpcb**) and (**1,4-di-I-tFb**)·(**2,2'-tpcb**) converged to their respective ratios after each was identified in the difference map and freely refined. Figures of all structures were rendered in the CCDC Mercury[1] software suite.

Table S1. Crystal data and structure refinement statistics for 2(**1,3-di-I-tFb**)·2(**2,2'-bpe**).

CCDC deposition number	1940759
Empirical formula	$C_{18}H_{10}F_4I_2N_2$
Formula weight/g·mol^{-1}	584.08
Temperature/K	296.15
Crystal system	orthorhombic
Space group	$Pna2_1$
a/Å	21.975(2)
b/Å	19.2247(19)
c/Å	9.0190(9)
α/°	90
β/°	90
γ/°	90
Volume/Å^3	3810.2(6)
Z	8
ρ_{calc}/g·cm^{-3}	2.036
μ/mm^{-1}	3.342
F(000)	2192
Crystal size/mm^3	0.32 × 0.23 × 0.22
Radiation	MoKα (λ = 0.71073)
2Θ range for data collection/°	3.706 to 53.308
Index ranges	$-27 \le h \le 27$, $-24 \le k \le 23$, $-11 \le l \le 11$
Reflections collected	24085
Independent reflections	7967 [R_{int} = 0.0302, R_{sigma} = 0.0287]
Data/restraints/parameters	7967/1/482
Goodness-of-fit on F^2	1.091
Final R indices [$I \ge 2\sigma (I)$]	R_1 = 0.0537
	wR_2 = 0.1355
R indices (all data)	R_1 = 0.0685
	wR_2 = 0.1522
Largest diff. peak/hole/e·Å^{-3}	3.50/-1.09
Flack parameter	0.284(14)

Table S2. Crystal data and structure refinement statistics for **2(1,3-di-I-tFb)**·(**2,2'-tpcb**).

CCDC deposition number	1967637
Empirical formula	$C_{36}H_{20}F_8I_4N_4$
Formula weight/g·mol^{-1}	1168.16
Temperature/K	150.15
Crystal system	monoclinic
Space group	$P2_1/c$
a/Å	9.3925(9)
b/Å	18.8849(19)
c/Å	20.596(2)
α/°	90
β/°	90.337(5)
γ/°	90
Volume/Å^3	3653.2(6)
Z	4
ρ_{calc}/g·cm^{-3}	2.124
μ/mm^{-1}	3.486
F(000)	2192
Crystal size/mm^3	0.205 × 0.145 × 0.03
Radiation	MoKα (λ = 0.71073)
2Θ range for data collection/°	4.844 to 52.798
Index ranges	$-11 \leq h \leq 11$, $-23 \leq k \leq 23$, $-25 \leq l \leq 25$
Reflections collected	41568
Independent reflections	7413 [R_{int} = 0.0373, R_{sigma} = 0.0299]
Data/restraints/parameters	7413/4/498
Goodness-of-fit on F^2	1.153
Final R indices [$I \geq 2\sigma(I)$]	R_1 = 0.0463
	wR_2 = 0.0864
R indices (all data)	R_1 = 0.0613
	wR_2 = 0.0926
Largest diff. peak/hole/e·Å^{-3}	3.41/-3.00
Flack parameter	-

Table S3. Crystal data and structure refinement statistics for (**1,4-di-I-tFb**)·(**2,2'-bpe**).

CCDC deposition number	1940762
Empirical formula	$C_{18}H_{10}F_4I_2N_2$
Formula weight/g·mol^{-1}	584.08
Temperature/K	296.15
Crystal system	tetragonal
Space group	$I4_1cd$
a/Å	14.1404(14)
b/Å	14.1404(14)
c/Å	38.138(4)
α/°	90
β/°	90
γ/°	90
Volume/Å^3	7625.7(17)
Z	16
ρ_{calc}/g·cm^{-3}	2.035
μ/mm^{-1}	3.34
F(000)	4384
Crystal size/mm^3	0.23 × 0.19 × 0.045
Radiation	MoKα (λ = 0.71073)
2Θ range for data collection/°	5.762 to 53.438
Index ranges	-17 ≤ h ≤ 17, -17 ≤ k ≤ 17, -48 ≤ l ≤ 41
Reflections collected	21996
Independent reflections	3771 [R_{int} = 0.0374, R_{sigma} = 0.0225]
Data/restraints/parameters	3771/1/242
Goodness-of-fit on F^2	1.062
Final R indices [$I \geq 2\sigma (I)$]	R_1 = 0.0260
	wR_2 = 0.0552
R indices (all data)	R_1 = 0.0394
	wR_2 = 0.0603
Largest diff. peak/hole/e·Å^{-3}	0.24/-0.34
Flack parameter	0.254(13)

Table S4. Crystal data and structure refinement statistics for (**1,4-di-I-tFb**)·(**2,2'-tpcb**).

CCDC deposition number	1967634
Empirical formula	$C_{30}H_{20}F_4I_2N_4$
Formula weight/g·mol^{-1}	766.3
Temperature/K	296.15
Crystal system	monoclinic
Space group	$C2/c$
a/Å	25.422(3)
b/Å	5.4525(5)
c/Å	21.183(2)
α/°	90
β/°	112.099(5)
γ/°	90
Volume/Å^3	2720.5(5)
Z	4
ρ_{calc}/g·cm^{-3}	1.871
μ/mm^{-1}	2.367
F(000)	1480
Crystal size/mm^3	0.16 × 0.16 × 0.05
Radiation	MoKα (λ = 0.71073)
2Θ range for data collection/°	6.326 to 53.426
Index ranges	-32 ≤ h ≤ 32, -6 ≤ k ≤ 6, -26 ≤ l ≤ 21
Reflections collected	7854
Independent reflections	2870 [R_{int} = 0.0338, R_{sigma} = 0.0413]
Data/restraints/parameters	2870/1/196
Goodness-of-fit on F^2	1.077
Final R indices [I ≥ 2σ (I)]	R_1 = 0.0352
	wR_2 = 0.0745
R indices (all data)	R_1 = 0.0641
	wR_2 = 0.0838
Largest diff. peak/hole/e·Å^{-3}	0.441/-0.676
Flack parameter	-

S5. Bond Metrics and Melting Point Tables.

Table S5. Bond metrics for cocrystals.

Cocrystal	X-Bond	*d* (N···I)/Å	*Θ* (C-I···N)/°	X-Bond Type	Prs*
2(**1,3-di-I-tFb**)·(**2,2′-bpe**)	I1···N1	2.96(2)	172.64	I	16
	I2···N2	3.00(1)	174.16	I	15
	I3···N3	2.99(1)	172.24	I	15
	I4···N4	3.04(1)	174.42	I	14
2(**1,3-di-I-tFb**)·(**2,2′-tpcb**)	I1···N1	2.949(6)	174.39	I	16
	I2···N4	3.009(6)	167.06	I	15
	I3···N3	2.947(6)	177.44	I	17
	I4···N2	3.131(6)	169.48	I	11
(**1,4-di-I-tFb**)·(**2,2′-bpe**)	I1···N1	2.967(8)	177.09	I	16
	I2···N2	2.982(8)	177.16	I	16
(**1,4-di-I-tFb**)·(**2,2′-tpcb**)	I1···N1A	3.064(4)	174.36	I	13
	I1···N1B	2.942(4)	171.42	I	17

* prs ≡ percent relative shortening = {1 - d(N···I)/[r_{vdW}(I) + r_{vdW}(N)]}·100, with r_{vdW}(N) = 1.55 Å and r_{vdW}(I) = 1.98 Å.

Table S6. Melting point data for cocrystals and coformers thereof.

**Compound**	**mp/°C**	**Solvent**	**Reference**
**2,2'-bpe**	119-120	Et_2O	*J. Org. Chem.* **1956,** *21,* 1420-1422
**1,3-di-I-tFb**	-*	-	-
**1,4-di-I-tFb**	108-110	CCl_4	*Chem. Eur. J.* **2003,** *9,* 3974-3983
2(**1,3-di-I-tFb**)·(**2,2'-bpe**)	90-91	$CHCl_3$	This work
2(**1,3-di-I-tFb**)·(**2,2'-tpcb**)	112-117	$CHCl_3$	This work
(**1,4-di-I-tFb**)·(**2,2'-bpe**)	155-156	$CHCl_3$	This work
2(**1,4-di-I-tFb**)·(**2,2'-tpcb**)	174-175	$CHCl_3$	This work

* liquid at SATP

S6. References

[1] C. F. Macrae, I. J. Bruno, J. A. Chisholm, P. R. Edgington, P. McCabe, E. Pidcock, L. Rodriguez-Monge, R. Taylor, J. van de Streek, P. A. Wood, *J. Appl. Cryst.* **2008**, *41*, 466-470.
[2] Z. Otwinowski,W. Minor, *Methods Enzymol.* **1997**, *276*, 307-326.
[3] G. M. Sheldrick, *Acta Cryst.* **2015**, *A71*, 3-8.
[4] G. M. Sheldrick, Acta *Cryst.* **2015**, *C71*, 3-8.
[5] O. V. Dolomanov, L. J. Bourhis, R. J. Gildea, J. A. K. Howard, H. Puschmann, *J. Appl. Cryst.* **2009**, *42*, 339-341.
[6] A. L. Spek, *Acta Cryst.* **2009**, *D65*, 148–155.
